# 2564 Designing for dissemination: Characteristics of Clinical and Translational Science Award (CTSA) hubs as adopters of clinical and translational science innovation

**DOI:** 10.1017/cts.2018.61

**Published:** 2018-11-21

**Authors:** Elaine H. Morrato, Lindsay Lennox, Anne Schuster

**Affiliations:** 1 Colorado School of Public Health, University of Colorado, Anschutz Medical Campus, Aurora, CO, USA; 2 Department of Communication, University of Colorado, Denver, CO, USA

## Abstract

OBJECTIVES/SPECIFIC AIMS: The Clinical and Translational Science Award (CTSA) program is a national consortium of 50+ academic medical research centers charged with accelerating the translation of clinical research. In 2017, the NIH National Center for Advancing Translational Sciences anticipates total CTSA program funding of over $500M. The consortium’s hub-and-spoke structure makes it a natural dissemination network, and the newest funding announcement makes dissemination of innovation across the consortium an explicit goal, but characteristics of CTSA hubs as adopters and transmitters of innovation are unknown. METHODS/STUDY POPULATION: A content analysis was conducted using data from CTSA hub Web sites (n=64) and a structured coding taxonomy based on 6 constructs drawn from literature about diffusion of innovation in service organizations (Greenhalgh *et al.*, 2004): dissemination priority, institutional complexity, communication infrastructure, support for dissemination/implementation functions, cross-institutional collaboration/networking, and leadership composition. RESULTS/ANTICIPATED RESULTS: In total, 52% of hubs will renew under the new PAR in the next few years, providing an incentive to demonstrate dissemination capacity (although hubs will likely lag in operationalizing these activities until they are funded). A third of hubs (34%) represent more than one academic/research institution, and almost 80% of hubs have more than one clinical affiliate. To accommodate these different levels of institutional complexity, broad diffusion will require multi-modal, locally adapted dissemination efforts. Only 25% of hubs have capacity to undertake additional dissemination activities, and only 27% provide formal D&I support, suggesting that additional capacity/support will be needed to operationalize the CTSA dissemination mission. In total, 30% of hubs participate in cross-institutional collaboration/networking, so many may not have existing norms/tools supporting inter-institutional collaboration, but 77% include leadership from outside the School of Medicine, facilitating effective intrainstitutional dissemination. DISCUSSION/SIGNIFICANCE OF IMPACT: Understanding more about CTSA hubs as both adopters and transmitters of innovation can facilitate strategic use of these sites as a built-in dissemination network to amplify the reach and impact of clinical innovation and improve population health. Based on this initial analysis, the CTSA network does not appear to be fully primed for broad, rapid dissemination of innovation across its sites. In-depth interviews are being conducted to investigate CTSA hubs’ perceptions of their dissemination capacity and roles as adopters and transmitters of innovation.

